# Integrated enhanced cognitive behavioural (I-CBTE) therapy significantly improves effectiveness of inpatient treatment of anorexia nervosa in real life settings

**DOI:** 10.1186/s40337-022-00620-y

**Published:** 2022-07-08

**Authors:** Ali Ibrahim, Sharon Ryan, David Viljoen, Ellen Tutisani, Lucy Gardner, Lorna Collins, Agnes Ayton

**Affiliations:** 1 Oxford Health NHS Foundation Trust, Oxford, UK; 2 Department of Psychiatry, University of Oxford, Oxford, UK; 3 Arts and Sciences, University College London, London, UK

**Keywords:** Anorexia nervosa, Inpatient, Treatment, Cognitive behavioural therapy, Longitudinal cohort study

## Abstract

**Background:**

Inpatient treatment of anorexia nervosa can be lifesaving but is associated with high rates of relapse and poor outcomes. To address this, the Oxford service has adapted the enhanced cognitive behavioural treatment (CBTE) model, first developed for inpatients in Italy to a UK national health service (NHS) setting. In this study, we compared the outcomes from treatment as usual (TAU), integrated CBTE (I-CBTE), and alternative treatment models in routine UK clinical practice.

**Methods:**

This is a longitudinal cohort study, using routinely collected data between 2017 and 2020 involving all adults with anorexia nervosa admitted to specialist units from a large geographical area in England covering a total population of 3.5 million. We compared TAU with (1) I-CBTE (13 weeks inpatient CBTE, restoration to a healthy weight, combined with 7 weeks day treatment followed by 20 weeks of outpatient CBTE; (2) standalone inpatient CBTE (due to insufficient resources since the pandemic; and (3) 6–8 weeks admission with partial weight restoration as crisis management. Primary outcome measures (min. 1 year after discharge from hospital) were defined as: (1) good outcome: Body Mass Index (BMI) > 19.5 and no abnormal eating or compensatory behaviours; (2) poor outcome: BMI < 19.5 and/or ongoing eating disorder behaviours; (3) readmission; or (4) deceased. Secondary outcomes were BMI on discharge, and length of stay.

**Results:**

212 patients were admitted to 15 specialist units in the UK depending on bed availability. The mean age was 28.9 (18–60) years, mean admission BMI was 14.1 (10–18.3), 80% were voluntary. At minimum 1-year follow up after discharge, 70% of patients receiving I-CBTE and 29% standalone inpatient CBTE maintained good outcomes, in contrast with < 5% TAU and crisis management admission. Readmission rates of I-CBTE were 14.3% vs ~ 50% (χ^2^ < 0.0001) in the other groups. The main predictors of good outcome were reaching healthy BMI by discharge, I-CBTE and voluntary status. Age, psychiatric comorbidity and length of stay did not predict outcomes. BMI on discharge and length of stay were significantly better in the CBTE groups than in TAU.

**Conclusions:**

Our main finding is that in a real-life setting, I-CBTE has superior short- and minimum 1 year outcomes as compared with alternative inpatient treatment models. Dissemination of I-CBTE across the care pathway has the potential to transform outcomes of inpatient treatment for this high-risk patient population and reduce personal and societal costs.

**Supplementary Information:**

The online version contains supplementary material available at 10.1186/s40337-022-00620-y.

## Plain English summary

Outcomes for adults requiring inpatient treatment for anorexia nervosa are poor. The aim of this work was to evaluate a recently introduced Integrated Cognitive Behavioural Therapy (I-CBTE) in Oxford, adapted from a model first developed for inpatients in Italy, compared with alternative inpatient treatment programmes in the UK. I-CBTE is an innovative approach which combines a time limited, planned admission of 13-weeks with the goal of full weight restoration, 7-weeks day treatment and ongoing outpatient CBTE. Treatment as usual includes an eclectic multidisciplinary approach, which is often unplanned and poorly coordinated across the care pathway in routine practice.

Between 2017 and 2020, we systematically analysed routinely collected data for patients admitted to 15 specialist units from a population of 3.5million in England. We looked at outcomes between admission and discharge, and at one year follow up. 70% of patients receiving I-CBTE, maintained healthy weight at one year after discharge from hospital (without binging or purging), in contrast with less than 5% of those in alternative programmes that result in partial weight restoration. Readmission rates were 14.3% and ~ 50% respectively. Partial weight restoration resulted in high readmission rates and therefore should be discouraged.

Our findings show that continuity and consistency of the I-CBTE approach are fundamental for maintaining good outcomes.

**Supplementary Information:**

The online version contains supplementary material available at 10.1186/s40337-022-00620-y.

## Background

Anorexia nervosa is a difficult to treat mental disorder with high rates of physical and psychiatric morbidities and mortality [1]. International guidelines agree that outpatient psychological treatment should be the first line intervention [2, 3]. However, regardless of the treatment model, not everyone responds to outpatient treatment [4, 5], and a significant proportion of patients remain chronically ill or require more intensive treatment [6, 7]. The evidence base for inpatient treatment is weak, and consequently there are significant international variations in practices [8, 9]. The availability of specialist inpatient treatment is dependent on national guidelines and funding arrangements in each country which often results in poor access and a crisis for patients and families [10].

In the UK, admission tends to be the last resort for patients whose physical health is severely compromised. Hospital admissions in England of people with eating disorders have increased from 4,849 in 2007/8 to 23,954 in 2020/21 [11]. Approximately 70% of these are adults. In the UK, National Health Service England (NHSE) commissions 455 specialist adult eating disorder beds. Approximately half of these are provided by the independent sector, and the remaining by multiple NHS providers. The independent hospitals are free at the point of delivery. Owing to the shortage of specialist beds in the UK, many patients have to wait for admission until they are gravely ill. This situation has worsened since the pandemic [12] and may explain an average length of stay that is longer than in other countries [13–16].

Cohort studies consistently show that while most patients gain weight in a hospital setting [14, 17, 18], the core eating disorder psychopathology often remains unresolved, and outcomes are unsatisfactory. In the UK, the majority of adult patients are discharged without reaching a healthy weight [13, 14], and there are high relapse and mortality rates [19–21]. However, the poor outcomes can be viewed as a chicken and egg dilemma. If only the most severely ill patients are admitted to hospital, it should not be surprising that recovery rates are lower than for those responding to outpatient treatment alone. While there is increasing recognition that early intervention has better outcomes [22], this principle has not been applied to inpatient treatment. Most adults requiring specialist inpatient treatment have been ill for years, and many have had several previous hospitalisations. The needs of this patient population should not be neglected, as they have the highest risk of premature mortality [20]. It is possible that restricting admissions to those, who are severely compromised, and discharging patients at a low weight contributes to poor outcomes and causes harm [23, 24].

### Current inpatient practices in the UK: treatment as usual (TAU)

International guidelines concerning the optimal inpatient treatment models vary [3, 8] owing to the limited evidence base. Current UK inpatient treatment programmes broadly follow the NHSE Standard Contract for Specialised Eating Disorder services and the NICE guidelines [2, 25]. These programmes include an eclectic combination of multidisciplinary interventions, including weight restoration, group and individual therapies of various modalities. Both NHS and independent providers commissioned by NHSE are required to meet the standards in the contract.

The NHSE contract recommends three broad types of admissions [25]:

1. Urgent/unplanned admissions with ‘modest weight restoration’
2. Planned short term admission for ‘medical stabilisation’ or symptom interruption
3. Symptom recovery admissions: weight restoration to “normal weight or weight at which patient can reliably continue independent weight restoration/ weight maintenance with less intensive input” and improved eating behaviours and psychological understanding.

In routine practice, these three options are rarely distinct. The NHSE contract is based on consensus rather than robust evidence. The multiple recommended psychological interventions were developed independently for outpatients and have never been tested in combination in inpatient settings. The potential weakness of these programmes is that there is often a risk of giving conflicting messages to the patient [26] and this may explain why disengagement and self-discharge are common—as much as 60% in some studies [27] The content of TAU differs across inpatient units and even across time within the same unit. Furthermore, unplanned admissions are common, and the majority of patients are discharged without reaching a healthy weight [28]. So, it should not be entirely surprising that despite the implementation of the national contract, outcomes of inpatient treatment remain poor, and that the number of people requiring hospitalisation with eating disorders has been increasing, partially owing to readmissions [29].

### Transition and care coordination

Although UK guidelines recommend clear care planning in preparation for admission and for discharge [2, 30], there is limited guidance as to the details of how this should be implemented. Most patients experience unplanned admissions and interruptions in therapy, as well as changes in therapeutic models, before and after admission, as inpatient and community teams are provided by separately commissioned teams.

### Recent changes in funding arrangements

In England, funding arrangements have shifted from NHSE commissioning to regional NHS collaborations, with the intention of transforming care pathways for the local populations [31]. In 2018, the Healthy Outcomes for People with Eating Disorders Provider Collaborative (HOPE PC) was established, including 5 NHS organisations in Oxfordshire, Buckinghamshire, Wiltshire, Berkshire and Gloucestershire. The total population of the geographical area is 3.5 million. The main goal of the network was to bring together several organisations providing specialist inpatient and community services for adults with eating disorders and equitable access to inpatient treatment through a single point of access for referrals [32]. The inpatient beds are available in the Oxford and Marlborough NHS units, and the independent Priory Group. The Priory has a national chain of specialist eating disorder units, which are commissioned by the NHS.

The PC agreed to monitor and compare outcomes of all patients who were admitted to the different providers.

### The adaptation of intensive enhanced cognitive behavioural therapy (CBTE) approach in Oxford

Over the last 15 years, Dalle Grave’s team in Italy, in collaboration with Fairburn in Oxford, has adapted CBTE, which had been originally developed as one-to-one outpatient therapy [33], to a new, whole-team, stepped care treatment programme for people with severe eating disorders requiring intensive treatment [26, 34, 35]. The novelty of this programme is the clear theoretical underpinning of treatment, and continuity of evidence based psychological treatment throughout the inpatient, day patient and community pathways.

The intensive CBTE treatment fosters therapeutic optimism, and has four main goals:

1. To engage patients in the treatment and involve them actively in the process of change;
2. To remove the eating disorder psychopathology, i.e., dietary restraint and restriction (and reverse malnutrition), extreme weight-control behaviours, and preoccupation with shape, weight and eating;
3. To correct the mechanisms maintaining the eating disorder psychopathology;
4. To ensure lasting change.

For full details, we refer to the published literature, including two manuals [26, 34] that describe the method. In summary: the Integrated CBTE treatment programme is time limited to 13 weeks inpatient treatment, followed by 7-weeks day treatment for stabilisation of healthy weight and ongoing outpatient CBTE afterwards lasting for 40 weeks in total, (in line with the length of treatment recommended by NICE) [2].

Given the differences between the health care systems and legal frameworks of the UK and Italy, we had to adjust the programme for the NHS. Dalle Grave’s unit in Italy is an independent hospital that only admits patients who, after preparation, consent to the full programme, while NHS services are required to admit patients who consent only to partial weight restoration or who require compulsory admission and treatment.

As a result, we introduced two pathways:

1. I-CBTE: full weight restoration with time limited admission (12–13 weeks) and 7 weeks stepped down day treatment, followed by outpatient CBTE replicating Dalle Grave’s model.
2. 6–8 weeks ‘Crisis management’ admission for those patients who do not consent to I-CBTE but are not detainable. This was due to having to comply with the NHSE contract that includes planned short-term admissions.
  Detained patients were encouraged to choose full weight restoration and were offered the whole programme.
3. The impact of the pandemic on the implementation of I-CBTE and TAU:
  Unfortunately, since the onset of the pandemic, most day services have closed or have had to run with reduced capacity due to infection control and staffing challenges. Patients have been unable to access ongoing individual psychological therapy after discharge and have been placed on lengthy waiting lists for therapy.
  Despite these challenges, the Oxford inpatient team maintained the key multidisciplinary components of CBTE. This situation has created a natural experiment to compare the standalone inpatient CBTE with the I-CBTE integrated stepped care model, and we included this group in our analysis.
  TAU has not changed as inpatient treatment is traditionally provided separately from outpatient treatment in the UK
  The transformation of the Oxford service from a TAU to the I-CBTE treatment programme included the following changes.

#### Preparation for admission

We introduced a multidisciplinary/multiagency admission planning meeting for all patients regardless of severity or legal status. Previously, most admissions were unplanned and we had high rates of self-discharge. Preparation for admission is fundamental for successful inpatient treatment, even for the physically compromised patient [26]. Ideally, patients should start psychological treatment before admission.

The purpose of this meeting is to help the person consider treatment options, the benefits and risks related to both options and to encourage them to overcome their eating disorder. This approach aims to empower the patient: encouraging a sense of control at a time when feelings of loss of control are common and act as a barrier to accepting care; and fostering a sense of autonomy as well as therapeutic alliance, collaboration and developing trust with the inpatient team. The multidisciplinary team introduces the treatment programme on the unit and helps the patient to make an informed decision from the two time-limited options.

The treatment team always strongly encourages the patient to sign up for the complete I-CBTE programme, explaining that research shows higher likelihood of recovery. In our experience, patients and carers value this evidence-based information, which helps them make informed decisions, even if they are highly ambivalent about the admission.

Typically, the discharge date is agreed before admission. This helps all parties to remain focussed on admission goals and to plan for continuity of treatment after discharge. The patient and the carers have an opportunity to visit the unit and receive written information to help familiarise themselves with the treatment available. This is essential for managing anxiety and to start therapeutic engagement.

Dalle Grave recommends several sessions for this engagement; however, due to resource limitations, we have only been able to offer one meeting for most patients. Feedback has been positive, although some clearly require more than one session of preparation to sign up for full treatment, and we are planning to address this in the next steps of service development. Since the pandemic, these meetings have been remote. The technology allowed the inclusion of multiple stakeholders, including the family, community teams, and GP/other agencies.

#### ‘Crisis management’ admission

In Oxford, we introduced the term ‘crisis management’ for planned 6–8 weeks admissions with partial weight restoration. This was to meet NHSE contract requirements. The name was changed from medical stabilisation to prevent inadvertently reinforcing the psychopathology, and misleading patients and carers, who often interpret ‘medical stabilisation’ literally. If someone is discharged when still malnourished, they may be over an immediate crisis, but are not stable, as chronic malnutrition is progressively harmful. The goal is to help the patient introduce regular eating, behavioural changes and reach a BMI of minimum 16, or 6-8 kg of weight gain and prepare for further outpatient treatment. Intensive aftercare is recommended to continue with progress. Patients who choose the short admission can opt to full I-CBTE if they change their minds during admission.

#### Rate of weight restoration

We also reviewed the weight restoration programme. The rate of expected weight gain in the UK is 0.5–1 kg per week in hospital setting [2]. In the past, we also followed this practice and used very sensitive medical grade scales on the unit, which recorded minuscule changes in weight. However, this inadvertently reinforced the patient’s preoccupation with minor details. We changed to 0.5 kg scale accuracy and faster weight restoration (1–1.5 kg/week) following Dalle Grave recommendation [26]. Both changes were well received when the rationale was explained, and they improved the rate of weight restoration and reduced length of stay. Collaborative weighing and interpretation of the weight graphs are an important part of CBTE. It is essential that the patient understands the need for weight restoration: malnutrition is one of the principal maintaining factors of anorexia nervosa [33]. With psychological treatment, most patients recognise that reversal of malnutrition is a necessary step towards recovery. The dietetic team was crucial in implementing these changes, helping to manage the patients’ distress in the dining room using quality improvement methodology [36].

#### Psychological treatment

In addition to individual CBTE, we developed a rolling 20-week multidisciplinary group programme following CBTE principles. The individualised CBTE formulation for every patient considers all maintaining factors (e.g., physical and mental health co-morbidities, individual strengths and difficulties, and social factors) and guides the role of each team member as to how best to support the patient. All members of the team work in a co-ordinated and collaborative approach with the patient to address their individual maintaining factors through the setting of weekly goals and the ongoing development of new skills and strategies based on their formulations.

#### Transitions

Prior to the pandemic, we were able to deliver a 7-week day-programme and ongoing individual psychological treatment for patients on the I-CBTE pathway after discharge. People on the crisis management pathway received supportive management after discharge in their respective community teams.

## Aims

In this paper, we aim to compare short- and medium to long-term (as defined by minimum 1-year post discharge) outcomes of patients admitted to specialist inpatient units with anorexia nervosa from the HOPE PC geographical area using routinely collected data. The research questions were as follows:

1. Are there differences among TAU, I-CBTE, standalone inpatient CBTE and crisis management admissions in terms of post discharge outcomes?
2. Is there a difference among the four different treatment programmes in terms of length of stay and Body Mass Index (BMI) on discharge?

The null hypothesis was that there would be no difference among different inpatient treatment approaches.

## Methods

**Fig. 1:**
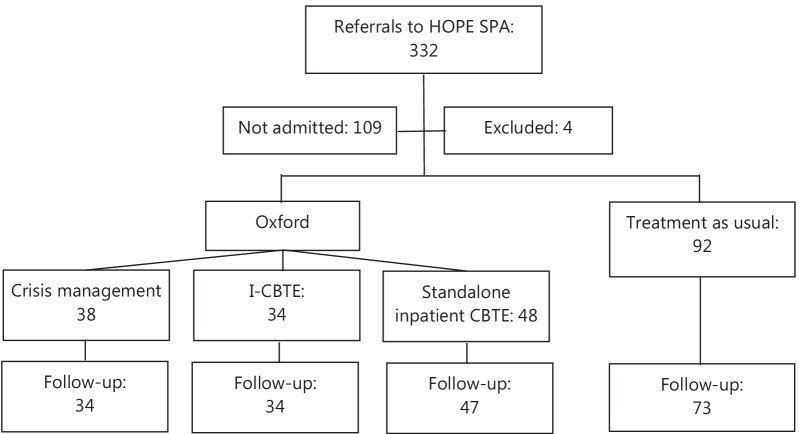
Flow diagram of analysis

This is a longitudinal cohort study, including all adults with anorexia nervosa admitted from the HOPE PC geographical area. Referrals and outcomes have been systematically monitored since the establishment of the PC. All referrals are discussed by senior clinicians representing each service at a weekly single point of access meeting, and patients are allocated based on bed availability, urgency as well as proximity to home. Approximately two third of the patients were admitted (Fig. 1). Waiting times for admissions were between 1–2 months. Patients, were not admitted if they did not consent to admission and their community team felt that admission was no longer necessary. Data has not been collected on patients not admitted. Patients admitted to Oxford were offered I-CBTE, but could opt for 6–8 weeks ‘crisis management’ admission with partial weight restoration if they did not consent to weight restoration to a healthy weight. Detained patients were encouraged to engage with I-CBTE. During the pandemic, day and outpatient services were disrupted, as described above, hence only standalone inpatient CBTE could be provided. Treatment as usual (TAU) continued as normal elsewhere. All units took detained patients.

Routine data collection included demographics, ICD-11 diagnosis, main categories of comorbidties, legal status, BMI on admission at discharge, and minimum 1-year outcome after discharge from hospital. This provided an opportunity for comparison among different models of inpatient treatment and type of aftercare (Fig. 1).

Inclusion criteria: all patients with an ICD-11 diagnosis of anorexia nervosa and related disorders who were admitted to a specialist inpatient unit following a referral to the HOPE PC single point of access between 2018- and December 2020, and all patients who were offered I-CBTE since its introduction in 2017. All patients included in the study had a BMI below 18.5 on admission.

Patients who were older than 60 years of age were not included in the analysis.

We compared TAU with the Oxford service, which offered I-CBTE, planned 6–8 weeks crisis management admission for the non-consenting voluntary patients, and the standalone inpatient CBTE since the pandemic. Detained patients, who did not engage with aftercare, were included in the standalone inpatient CBTE group for analysis.

Categorical outcomes at minimum 1 year after discharge from hospital were used as the primary outcome measure;

1. Good outcome: the patient is within the healthy weight range (BMI > 19.5) with no abnormal eating or compensatory behaviours (based on their detailed electronic health records) and either discharged from outpatient services or completing treatment.
2. Poor outcome: patient remains BMI < 19.5 and/or ongoing eating disorder behaviours (regardless of whether still open to specialist services or discharged to primary care)
3. Readmission (any time after discharge).
4. Deceased (any time after discharge).

We decided to use a BMI of 19.5 as a cut-off point for definition of minimum healthy weight as opposed to a BMI of 18.5, which is the threshold for anorexia nervosa diagnosis, because maintaining a BMI of 18.5 for an adult Caucasian woman is unlikely without significant dietary restriction.

We additionally examined BMI on discharge and length of inpatient stay among different models, and predictors of good outcomes.

### Statistics

We analysed the data with SPSS 22, using descriptive statistics, Chi-squire test for comparing categorical variables, Independent T-test and ANOVA for continuous variables.

A stepwise linear regression was used to identify possible predictors of 1-year outcomes out of the following candidate variables: age, admission and discharge BMI, treatment approach, compulsory treatment, psychiatric comorbidities and length of stay. Correlation coefficients for each pair of variables were based on all the cases with valid data for that pair. Regression statistics are based on these correlations.

### Ethics

The study was approved by Oxford Health Foundation Trust Audit department as a service evaluation study. As only routinely collected data were used, there was no requirement for individual patient consent. All data were kept on a secure server.

## Results

### Demographic and clinical characteristics of the patients admitted to inpatient facilities

In total, 212 patients were admitted between 2017–2020 to 15 different specialist eating disorder units in England and Scotland: 120 to Oxford, 34 to Marlborough, 16 to the Priory within area, 29 to out-of-area independent units, and 13 to out-of-area NHS units. Patients were allocated for admission depending on bed availability and proximity to home.

The mean age was 28.9 years (17.1–59). Forty-seven percent of patients referred for admission were young adults between 17 and 25 years. Ninety-seven percent were female, and 99% had a primary diagnosis of ICD-11 anorexia nervosa, with the remaining patients having atypical presentation.

Table 1 shows baseline characteristics in the four treatment options. Patients who chose the crisis management pathway were significantly older. There were more detained patients in the standalone inpatient CBTE group.

**Table 1:** Characteristics of patients admitted to hospital from the HOPE geographical area

	Oxford Model			TAU	*P*
	I-CBTE	Standalone CBTE	Crisis management		
Age (years) mean ± SD	26.9 ± 9.6	28.6 ± 10.5	33.2 ± 10.9	27.0 ± 8.6	0.004
Female%	100%	100%	97%	97%	0.357
Compulsory admission %	17%	39%	5%	8%	0.00
BMI on admission mean ± SD	14.6 ± 1.3	14.5 ± 1.9	14.2 ± 1.6	14.6 ± 1.7	0.62
ICD-11 AN restrictive pattern	100%	92.2%	89.2%	89.3%	0.49
ICD-11 AN binge-purge pattern	0%	7.8%	10.8%	10.7%	
Psychiatric comorbidities					0.261
Depression	40%	21%	27%	29%	
Anxiety disorders (including PTSD, and obsessive–compulsive disorder)	16%	14%	18%	22%	
Personality disorder	4%	19%	9%	5%	
Autism spectrum disorder	8%	7%	27%	15%	
Other	8%	12%	3%	7%	

BMI: Body Mass Index; SD: standard deviation

There was no difference among the groups regarding the type of anorexia nervosa and main comorbidities, such as depressive disorders, anxiety disorders, autism spectrum disorder, or personality disorders.

Short term outcomes at discharge are summarised in Table 2. Discharge BMI was significantly higher in the CBTE groups than in TAU (BMI = 19.7 and 19 vs. 17), despite the shorter length of stay. Additional detail about different providers is included in Additional file 1: Table.

**Table 2:** Differences in short term outcomes among different treatment models (admission and discharge)

	Mean	95% confidence interval for mean		ANOVA *P*
		Lower bound	Upper bound	
Discharge BMI				
I-CBTE	19.7	19.4	20.0	< 0.0001
Standalone inpatient CBTE	19.0	18.5	19.5	
Crisis management	16.0	15.6	16.3	
TAU	17.0	16.6	17.4	
Length of stay (inpatient days only)				
I-CBTE	125.4	111.4	139.3	< 0.0001
Standalone inpatient CBTE	106.4	84.7	128.0	
Crisis management	50.1	39.6	60.7	
TAU	132.7	112.7	152.7	

I-CBTE: Full weight restoration, integrated CBTE, across inpatient, day patient and outpatient treatment

Standalone inpatient CBTE: Full weight restoration, inpatient CBTE, without consistent aftercare

Crisis management admission: 6–8 weeks planned admission with partial weight restoration, without consistent aftercare

TAU: treatment as usual: eclectic model, partial weight restoration without consistent aftercare

**Fig. 2:**
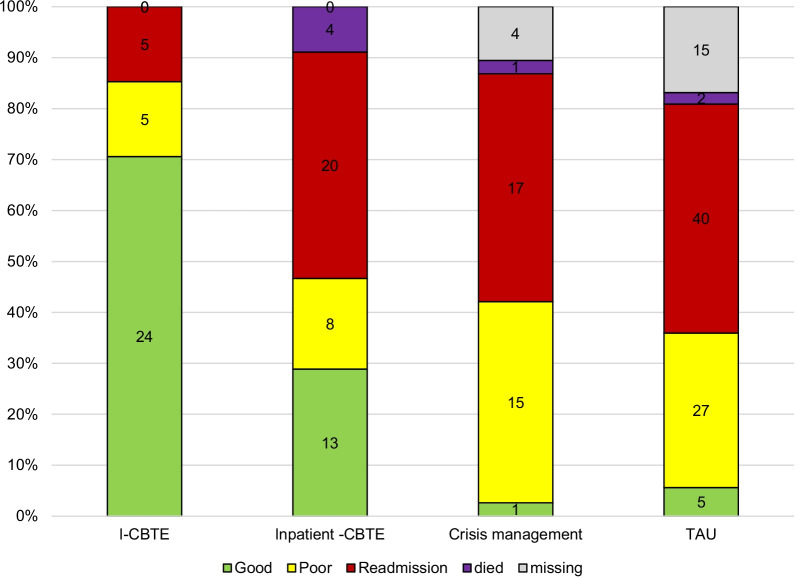
Outcome after discharge (minimum 1 year follow-up). Comparison of minimum 1-year outcomes after discharge among I-CBTE, standalone inpatient CBTE, Crisis management and TAU (Chi square < 0.0001)

Minimum 1-year outcome data were available for 188 (89%) patients. Follow-up information was unavailable for 21 participants (mostly students) who moved out of area and for another 4 who, after a lengthy hospitalization, were still within 12 months post discharge. I-CBTE was highly significantly better in helping people to maintain recovery over time (Fig. 2). Seventy percent of patients who received I-CBTE maintained good outcomes for a minimum of one year, while only 28% who received standalone inpatient CBTE and only 3–4% of those receiving crisis management admission or TAU achieved this (Chi square < 0.0001). This benefit was not dependent on age, comorbidity, or severity of malnutrition on admission. Patients with a good outcome reached a mean discharge BMI of 19.9 and continued with I-CBTE after discharge. Readmission rates was 14% in the I-CBTE group and between 38–44% in the others.

There was no significant difference between the outcomes of crisis admission as compared with TAU, despite the much longer length of stay in the latter. Approximately two third of patients remained chronically unwell in these groups.

Patient experience after 20 years of repeated admissions

I was admitted to the eating disorder unit at the Warneford Hospital in Oxford, in August 2017. I had been suffering from a complex, co-morbid eating disorder for nearly 2 decades; I was locked away in psychiatric institutions as a “treatment resistant” “revolving door patient”. The Oxford team provided a holistic, inclusive, ground-breaking and compassionate care with the I-CBTE model. I was able to work through all the issues and trauma of my long, terrible journey. For the first time ever, I reached a healthy weight. This helped me to think more clearly. I was encouraged to use my creativity, to imagine a new life. This was daunting, but exciting. On my discharge, I had support in the community, which helped me practice what I had learned in hospital and allowed my body to finish reaching its healthy weight. My illness was not really about weight, but learning to accept my body, whatever its weight, was essential. In the last 4 years, I have formed a completely new life for myself, which has nothing to do with eating disorders. This is the epitome of my recovery, the outcome of my I-CBTE treatment: I am successful, creative, needed. I will always be incredibly grateful to the multidisciplinary team in Oxford for their lifechanging care.

Stepwise linear regression showed that the main significant predictors of outcome were discharge BMI, I-CBTE and voluntary treatment, rather than age, admission BMI, length of stay, or psychiatric comorbidities (Table 3).

**Table 3:** Predictors of minimum 1-year outcome (Linear regression)

Model		Unstandardized Coefficients		Standardized Coefficients	t	Sig
		B	Std. Error	Beta		
1	(Constant)	5.092	0.588		9.03	< 0.0001
	Discharge BMI	− 0.159	0.033	− 0.43	− 5.168	< 0.0001
2	(Constant)	3.980	0.676		5.831	< 0.0001
	Discharge BMI	− 0.130	0.033	− 0.344	− 4.144	< 0.0001
	I-CBTE	0.274	0.089	0.29	3.491	0.0030
3	(Constant)	3.79	0.668		5.489	< 0.0001
	Discharge BMI	− 0.135	0.033	− 0.349	− 4.269	< 0.0001
	I-CBTE	0.271	0.088	0.299	3.661	0.003
	Legal status	0.215	0.089	0.178	2.28	0.017

Dependent variable: outcome after minimum 1 year following discharge from hospital

Excluded variables:

Model 1: I-CBTE, age, admission BMI, legal status, length of stay, and psychiatric comorbidities

Model 2: age, admission BMI, legal status, length of stay, and psychiatric comorbidities

Model 3: age, admission BMI, length of stay, and psychiatric comorbidities

Seven patients of the cohort (3%, aged 20–50 years) died following discharge from hospital. Three patients died within 6 months and four up to 4 years after discharge. All except one had a history of compulsory admission/treatment. All had significant comorbidities: one person had severe inflammatory bowel disease, one had obsessive compulsive disorder, three had alcohol or substance misuse, and four had previously attempted suicide. The cause of death was suicide in one case, two patients died of physical complications of the eating disorder, and four deaths were undetermined. All patients died during the pandemic when outpatient face to face treatment was disrupted. There were no deaths in the I-CBTE group, which again underlines the importance of integrated aftercare.

## Discussion

This is the first large UK systematic dataset comparing minimum 1 year post discharge outcomes of different inpatient treatment approaches for adults with anorexia nervosa.

Our main finding is that I-CBTE achieves significantly better outcomes as compared with alternative inpatient treatment models in real life settings. Seventy percent of patients who received I-CBTE during the whole care pathway achieved good outcomes at least one year after discharge from hospital, as compared with less than 5% of the patients in the TAU or crisis pathways. Standalone inpatient CBTE (without integrated aftercare) had intermediate outcomes. The main predictor of good outcome was I-CBTE, which included a combination of full weight restoration and a time-limited, step-care approach, with integrated CBTE throughout inpatient, day treatment and outpatient settings, without any interruption or gaps in treatment, and with support for the patient to address the maintaining factors of their eating disorder and to make lasting changes. Age, admission BMI, length of stay and psychiatric comorbidity did not predict outcomes. These findings give hope for people who have been chronically ill. Our results are consistent with previous studies of CBTE in severe and enduring anorexia nervosa [37, 38]. They are also consistent with previous findings, which showed that good medium to long-term outcome is related to reaching a healthy weight on discharge [23, 24, 39–41]. Our findings show that although weight restoration to a healthy weight range is essential for good outcomes, it is insufficient in itself: integrated CBTE through stepping down to day patient and ongoing individual psychological treatment is required to ensure that changes are lasting. When integrated aftercare was not possible, the standalone inpatient CBTE programme resulted in 28% good 1-year outcomes, but it had the same rates of readmission as TAU and crisis admission (43%). This shows that investment into integrated care is essential to reduce repeated admission rates.

These results are important replications of Dalle Grave’s findings [35, 38, 42] in routine NHS practice, despite the significant differences between the healthcare systems and staffing levels in England and Italy. Their unit only admits patients who, after preparation, consent to the full programme, whilst NHS specialist services are required to admit everyone who needs inpatient treatment, regardless of whether they consent to treatment or not. At the beginning of the adaptation of the I-CBTE model in Oxford there was considerable uncertainty as to whether patients who are detained would have a negative impact on the engagement and progress of others treated on the unit at the same time. While at times this needed careful management by the team, we found that individual admission planning, clear goal-oriented admission, and the consistent and integrated multidisciplinary approach helped patients to focus on their own treatment.

The biggest challenge to implementation in the NHS was an insufficient number of therapists to provide the integrated CBTE psychological treatment through the care pathway. Furthermore, the pandemic dramatically reduced the availability of day hospital provision. When integrated step-care was not achievable owing to insufficient resources, we found that although inpatient CBTE had better short-term outcomes than TAU, only 28% of patients were able to sustain good progress. This shows that integration and continuity of care without delay are essential for maintaining good outcomes after discharge.

The importance of managing transitions well and coordinating care between inpatient and community services is emphasised in multiple guidelines [8, 30, 43], but there are only a few studies exploring the effects of the quality and type of aftercare following discharge, and the impact of integration of psychological treatment across the care pathway.[44, 45]. The main strength of I-CBTE is the clarity and consistency of the treatment approach and the continuity of care when the patient is stepping down from inpatient treatment.

Our findings regarding outcomes of TAU replicate previous UK research. A 2013 multicentre cohort study of short-term outcomes of hospital treatment including 137 adults with anorexia nervosa, reported a mean admission BMI of 14, and discharge BMI of 17.3. Only 22% patients were discharged at BMI > 19 despite the lengthy admissions (average length of stay for inpatient treatment was 184 days and 126 days for day treatment) [14]. A study in Scotland reported similar results [13]. Our sample of 92 patients treated in 15 different units around the UK had similar outcomes: only a small proportion of patients were discharged at a BMI > 19.5 and less than 5% achieved good outcomes at one year after discharge. This suggests that there has been limited progress since 2013 in inpatient treatment of adults with anorexia nervosa in the UK, apart from shortening the mean length of stay [15].

We have clearly demonstrated that partial weight restoration programmes result in high readmission rates and poor outcomes regardless of the length of stay. Short term planned admissions and TAU had poor outcomes with low recovery rates and recurrent admissions. To our knowledge, there are no other medium to long-term outcome studies in the UK concerning short-term hospitalization or partial weight restoration. Short admissions are likely to be more effective if they are part of an integrated care programme. Trials in adolescent populations, such as short-term hospitalisation combined with family based treatment [46], or day hospital [47], showed positive results, which is consistent with our finding about the importance of ongoing integrated care after inpatient treatment. Unfortunately, gaps in service provision remain common and increase the risk of relapse [48].

The tragic death of patients in the cohort highlights the high risk of mortality in this patient population and is consistent with previous research [21, 49, 50]. All of them died during the pandemic, which inevitably had an impact on the aftercare arrangements in the relevant community services. Compulsory treatment, previous suicide attempts, and substance misuse have been associated with mortality, but further work is required to elicit modifiable risk factors for prevention.

### Strengths and limitations

We systematically analysed routine outcome data for patients admitted to 15 specialist units in England and Scotland in real life settings, and therefore believe that our findings are generalizable to UK practices.

There were a number of limitations. Data concerning eating disorder psychopathology were not systematically available and hence not included in this paper. Although shared outcome measures were agreed among HOPE PC partners, most units struggled to return data collection consistently. Consequently, we had to rely on the most commonly available robust categorical data. However, BMI and normalisation of weight is a good indicator of outcome in anorexia nervosa [39, 51]; and the rate of readmission and chronic malnutrition/ binge purging are robust outcomes and indicators of ongoing psychopathology. The latter information was available from the patient’s health records in specialist services, which is usually comprehensive regarding eating disorder psychopathology. We did not have access to primary care records for further follow-up for those patients who were discharged from secondary services, but the discharge letters were sufficiently detailed to help with categorising patients according to good or poor outcomes.

This was a longitudinal cohort study, and therefore the comparison of the different inpatient models was not based on randomisation. Patients were admitted based on bed availability and proximity and could choose I-CBTE or crisis management if admitted to Oxford. Randomised controlled trials of complex interventions for a high-risk population also have limitations [52]: as they rely on informed consent and therefore exclude the most complex patients and given the national shortage of beds in the UK, randomisation would not have been practicable. At baseline, there were more detained patients in the standalone CBTE programme and patients opting for ‘crisis management’ admission were older. Although we did not have consistent data on age of onset, most patients with anorexia have an onset in their adolescence or emerging adulthood, so the age of patients gives a good indication of the length of their illness.

It will be important to explore how to engage this more chronic patient group work towards recovery.

## Conclusions

We have shown that the I-CBTE model, which was first developed in Italy, is robust and is applicable to eating disorder services elsewhere. The treatment is highly collaborative, least restrictive, and treats the patients as adults. It supports them to work towards recovery and to develop autonomy based on their individual formulation, developing behavioural change and addressing their maintaining factors to achieve lasting changes.

The short- and medium to long-term outcomes of I-CBTE were significantly better than alternative models of inpatient treatment in real life settings. The key components of successful outcomes include engagement, preparation for admission, multidisciplinary working, full weight restoration, and gradual stepping down intensity with ongoing CBTE treatment.

### Recommendations

- The wider implementation of I-CBTE for treatment of people with anorexia nervosa, who require hospitalization could significantly improve outcomes. Training resources, such as manuals and online training are easily accessible and relevant to multidisciplinary teams to provide a cohesive approach.
- Funding arrangements need to align and support a stepped and integrated care pathway to achieve optimal outcomes. While there is national policy to support this in the UK, inpatient and community services are commissioned separately, so fragmentation of care is common and this needs to be addressed as a matter of priority.
- A national audit of inpatient treatment would help to improve transparency, develop the evidence base, and compare outcomes of different models. This should include monitoring admission and discharge parameters, length of stay, as well as transition of care and relapse rates. Similar calls have been made in the US [53] where there is a proliferation of for-profit organisations providing residential treatment. This is also essential in the UK where approximately 50% of beds are provided by the independent sector.
- A national register of deaths owing to eating disorders—similar to the National Confidential Inquiry [54]—would be important to reduce the risk of mortality in this patient population.
- Further research is needed on improving care pathways to help patients achieve sustained recovery after inpatient treatment [55]. The cost-effectiveness of integrated models of care as opposed TAU needs to be evaluated building on existing research [56].

## Supplementary Information

**Additional file 1: Table. **Admission and discharge parameters between different provider services**.**

## Data Availability

Available on request from the corresponding author.
