# Supplementary material for: Integrated enhanced cognitive behavioural (I-CBTE) therapy significantly improves effectiveness of inpatient treatment of anorexia nervosa in real life settings
Source: J Eat Disord. 2022 Jul 8;10:98. doi: 10.1186/s40337-022-00620-y (PMC9264571; doi:10.1186/s40337-022-00620-y)
Supplement: Supplementary file 1 — Additional file 1: Table. Admission and discharge parameters between different provider services. [file 40337_2022_620_MOESM1_ESM.docx]

**Supplementary Table: Admission and discharge parameters between different provider services**

|  | | N | Mean | 95% Confidence Interval for Mean | | ANOVA  P= |
| --- | --- | --- | --- | --- | --- | --- |
|  |  |  |  | Lower Bound | Upper Bound |  |
| Admission BMI | Oxford | 119 | 14.3 | 14.0 | 14.6 | 0.258 |
|  | Marlborough | 34 | 13.7 | 13.2 | 14.2 |  |
|  | Priory in area | 13 | 13.9 | 12.7 | 15.1 |  |
|  | independent OOA | 29 | 14.3 | 13.5 | 15.1 |  |
|  | NHS OOA | 12 | 13.5 | 12.0 | 15.0 |  |
|  | Total | 207 | 14.1 | 13.9 | 14.3 |  |
| Discharge BMI | Oxford | 119 | 18.2 | 17.8 | 18.5 | 0.001 |
|  | Marlborough | 34 | 17.1 | 16.5 | 17.7 |  |
|  | Priory in area | 12 | 17.2 | 16.2 | 18.1 |  |
|  | independent OOA | 27 | 17.4 | 16.4 | 18.3 |  |
|  | NHS OOA | 12 | 15.9 | 15.0 | 16.9 |  |
|  | Total | 204 | 17.7 | 17.4 | 18.0 |  |
| length of stay | Oxford | 119 | 95.4 | 83.6 | 107.1 | <0.0001 |
|  | Marlborough | 34 | 101.4 | 81.9 | 121.0 |  |
|  | Priory in area | 15 | 158.8 | 112.9 | 204.7 |  |
|  | independent OOA | 30 | 165.0 | 115.0 | 215.1 |  |
|  | NHS OOA | 13 | 109.8 | 73.4 | 146.3 |  |
|  | Total | 211 | 111.6 | 100.5 | 122.8 |  |
| Age on admission | Oxford | 117 | 29.3 | 27.3 | 31.2 | 0.175 |
|  | Marlborough | 34 | 25.9 | 23.1 | 28.7 |  |
|  | Priory in area | 16 | 25.2 | 21.0 | 29.4 |  |
|  | independent OOA | 30 | 29.7 | 25.9 | 33.5 |  |
|  | NHS OOA | 13 | 25.6 | 21.6 | 29.7 |  |
|  | Total | 210 | 28.2 | 26.9 | 29.6 |  |

Oxford: NHS service (including I-CBTE, Crisis admission and standalone inpatient CBTE)

Marlborough; In area NHS service, TAU

Priory and independent providers: TAU

NHS OOA: TAU

OOA: out of area
